# Rapid Water Softening with TEMPO-Oxidized/Phosphorylated Nanopapers

**DOI:** 10.3390/nano9020136

**Published:** 2019-01-22

**Authors:** Andreas Mautner, Thawanrat Kobkeatthawin, Florian Mayer, Christof Plessl, Selestina Gorgieva, Vanja Kokol, Alexander Bismarck

**Affiliations:** 1 Polymer & Composite Engineering (PaCE) Group, Institute of Materials Chemistry & Research, University of Vienna, 1090 Vienna, Austria; Thawanrat_KK@hotmail.com (T.K.); f.mayer@univie.ac.at (F.M.); alexander.bismarck@univie.ac.at (A.B.); 2 Polymer & Composite Engineering (PaCE) Group, Department of Chemical Engineering, Imperial College London, SW7 2AZ London, UK; 3 Department of Chemistry and Center of Excellence for Innovation in Chemistry, Faculty of Science, Prince of Songkla University, Songkhla 90110, Thailand; 4 Institute of Inorganic Chemistry, University of Vienna, 1090 Vienna, Austria; christof.plessl@univie.ac.at; 5 Institute for Engineering Materials and Design, Faculty of Mechanical Engineering, University of Maribor, 2000 Maribor, Slovenia; selestina.gorgieva@um.si (S.G.); vanja.kokol@um.si (V.K.)

**Keywords:** water hardness, nanocellulose, TEMPO-oxidation, phosphorylation, nanopaper, membrane

## Abstract

Water hardness not only constitutes a significant hazard for the functionality of water infrastructure but is also associated with health concerns. Commonly, water hardness is tackled with synthetic ion-exchange resins or membranes that have the drawbacks of requiring the awkward disposal of saturated materials and being based on fossil resources. In this work, we present a renewable nanopaper for the purpose of water softening prepared from phosphorylated TEMPO-oxidized cellulose nanofibrils (PT-CNF). Nanopapers were prepared from CNF suspensions in water (PT-CNF nanopapers) or low surface tension organic liquids (ethanol), named EPT-CNF nanopapers, respectively. Nanopaper preparation from ethanol resulted in a significantly increased porosity of the nanopapers enabling much higher permeances: more than 10,000× higher as compared to nanopapers from aqueous suspensions. The adsorption capacity for Ca^2+^ of nanopapers from aqueous suspensions was 17 mg g^−1^ and 5 mg g^−1^ for Mg^2+^; however, EPT-CNF nanopapers adsorbed more than 90 mg g^−1^ Ca^2+^ and almost 70 mg g^−1^ Mg^2+^. The higher adsorption capacity was a result of the increased accessibility of functional groups in the bulk of the nanopapers caused by the higher porosity of nanopapers prepared from ethanol. The combination of very high permeance and adsorption capacity constitutes a high overall performance of these nanopapers in water softening applications.

## 1. Introduction

Both calcium and magnesium ions, essential elements in the ecosystem required by animals and plants, are commonly present in natural water resources causing the permanent hardness of water [1]. Permanent water hardness is not subject to removal by heating, hence its name, and is defined as the sum of Ca^2+^ and Mg^2+^ expressed in terms of equivalent concentrations of CaCO_3_ [2,3]. High water hardness, typically above 14° dH, is an issue not only for the longevity of sanitary installations, such as boilers and within households, but also on an industrial scale. The generation of lime scale (CaCO_3_) results for example in blockage of pipes, membrane clogging, and reduction of the efficiency of heat exchangers, hence the inhibition of scale formation is of great importance [4]. Furthermore, high hardness water is claimed to be associated with health issues. For example, the disturbance of the skin barrier integrity and, consequently, atopic dermatitis have been identified to be potentially caused by high water hardness. Young infants are particularly at risk [5,6]. Therefore, the reduction of high water hardness (i.e., the removal of Ca^2+^ and Mg^2+^) is an important issue when controlling the quality of the water supply [7].

The task of water hardness removal, that is, water softening, is traditionally performed by means of discontinuously working ion-exchange materials [8,9]. Most prominently, zeolites are used for this purpose [7]. The use of zeolites for the exchange of divalent calcium and magnesium ions against monovalent sodium or potassium ions already dates back to the beginning of the last century [10,11,12]. In an effort to add efficiency to this customary methodology, the application of ultrasound during the ion-exchange process was proposed [13]. Zeolite-like materials for water softening were also generated from pumice stones [14,15], while other research groups utilized sulfonated plastic waste [16] or used polymer-enhanced porous carbon electrodes [17]. Electrochemical methods such as capacitive deionization [18], for example, by using graphene oxide [19], electrodeionization [20] as well as electrodialysis [21,22] are also considered promising water softening processes [23]. Recently, scale inhibition was also achieved with modified polyaspartic acid [24], poly(acrylic acid-co-allylpolyethoxy carboxylate) [25], or hydrophilic terpolymers containing carboxylic, sulfonic acid, and ether groups [26]. Furthermore, various types of membranes, in particular modified ultrafiltration or nanofiltration membranes, have been used for water softening [27]. Specifically, Bequet et al. used a photografting concept to modify a polysulfone ultrafiltration membrane with crosslinked polyacrylic acid [28]. Anim-Mensah et al. [29] and Lai et al. [30] used nanofiltration membranes, whereas Park et al. demonstrated an electro-membrane process [3]. More recently, Rajabzadeh et al. demonstrated layer-by-layer polyelectrolyte deposition on polyethersulfone hollow fiber ultrafiltration membranes [31], whereas Zhao et al. introduced a polyelectrolyte complex/carbon nanotube nanofiltration membrane for this purpose [32] and Zhang applied a positively charged capillary membrane with carbon-nanotubes [33]. All these examples had appreciable performance with regards to rejection or removal of calcium and magnesium ions. Recently, highly porous aerogels from renewable resources were also demonstrated to be efficient adsorption media for continuous metal adsorption with high adsorption capacity [34,35]. Given their high porosity and thus specific surface area, continuously operated adsorbers made from aerogels constitute a promising alternative in this regard.

Unfortunately, there are problems associated with these state-of-the-art processes. For batch-wise used adsorbents in particular, the disposal of saturated adsorbents and the discontinuous process constitute a drawback [36], whereas the drawback related to aerogels is the tedious preparation procedure: it is time- and energy-consuming to freeze and freeze–dry nanocellulose suspensions. Membranes are usually prepared from sophisticated, synthetic polymers via complex synthesis protocols which require many resources during their manufacture. Being derived from non-renewable resources, there is also the problem of the disposal of used membranes. However, the big advantage of membrane applications is the continuous process, including back-wash operations [37,38], but, unfortunately, reverse osmosis (RO) and nanofiltration (NF) membrane processes for desalination and water softening applications are very energy-consuming, requiring high driving pressures up to 50 bar [39]. Thus, a combination of those two approaches would be desirable: the application of an adsorbent material in a membrane process. Having this combination being based on materials from renewable resources would provide a sustainable solution in this domain.

Cellulose nanopapers, papers produced from cellulose nanofibrils (CNF) [40], have recently been applied to various membrane applications [41], both in size-exclusion operation [42,43,44,45] as well as for ion-exchange/adsorption nanopapers [46,47,48]. The pore size of size-exclusion nanopaper membranes was too big for the efficient removal of Ca^2+^ and Mg^2+^, and the adsorption capacity for metal ions only moderate at approximately 20 mg g^−1^. Moreover, the permeance of those nanopaper membranes was limited, being at least an order of magnitude off what would constitute an efficient process. Therefore, an approach is needed to maximize both adsorption capacity for metal ions and permeance. Permeances above 10,000 L h^−1^ m^−2^ MPa^−1^ would be appreciated with adsorption capacities for calcium and magnesium ions approaching 100 mg g^−1^.

In this study, we present a novel approach for the manufacture of high permeance, high affinity TEMPO-oxidized/phosphorylated nanopapers to remove Ca^2+^ and Mg^2+^ for water softening. We propose that key to the efficiency of nanopapers is the use of a low surface tension organic liquid for the preparation of the nanopapers. Accordingly, ethanol was chosen as suspension media instead of water. This was expected to enable the formation of a highly porous network [49]. Functional groups are present in the network and available for adsorption not only on the surface of the nanopaper but also in its bulk. This enables the efficient adsorption of cations. Furthermore, enhanced porosity is expected to give rise to very high permeances potentially making the nanopapers highly efficient.

## 2. Materials and Methods

### 2.1. Materials

2,2,6,6-Tetramethyl-1-piperidinyloxyl (TEMPO)-oxidized CNF (TO-CNF) with diameters of 3 to 5 nm and lengths of 500–2000 nm bearing 1.3 ± 0.1 mmol/g of carboxylic groups [50] were supplied by Betulium (Espoo, Finland). 1-Ethyl-3(3-dimethylaminopropyl)-1-carbodiimide hydrochloride (EDCHC), N-hydroxysuccinimide (NHS), 2-(N-morpholino) ethanesulfonic acid (NMESA), 3-aminopropylphosphoric acid (3APPA), CaCl_2_, MgCl_2_, KOH, NaOH, HCl, and KCl were purchased from Sigma-Aldrich (St. Louis, MO, USA). All chemicals were reagent grade and used as received without further purification. Milli-Q water was used at all stages for the preparation of the nanocellulose. For adsorption studies, deionized water was purified (18.2 MΩ cm) with an ELGA Purelab Classic (ELGA LabWater, Celle, Germany).

### 2.2. Preparation of CNF

#### 2.2.1. Preparation of Unmodified CNF

As reference, unmodified nanofibrils were prepared following a protocol previously reported [51]. The raw material was fiber sludge, a paper industry residue, which had a cellulose and hemicellulose content of 95 % and 4.75 %, respectively, kindly provided by Processum AB (Domsjö, Sweden). The raw material was immersed in distilled water at a consistency of 3 wt % for 2 h and then dispersed using a mechanical blender (Silverson L4RT, Chesham, Buckinghamshire, England) at 3000 rpm for 10 min. By grinding the suspension with an ultra-fine grinder (MKCA 6-3, Masuko Sangyo, Kawaguchi, Japan), unmodified nanofibrils (CNF) were obtained.

#### 2.2.2. Preparation of Phosphorylated TEMPO-CNF

**Scheme 1:**
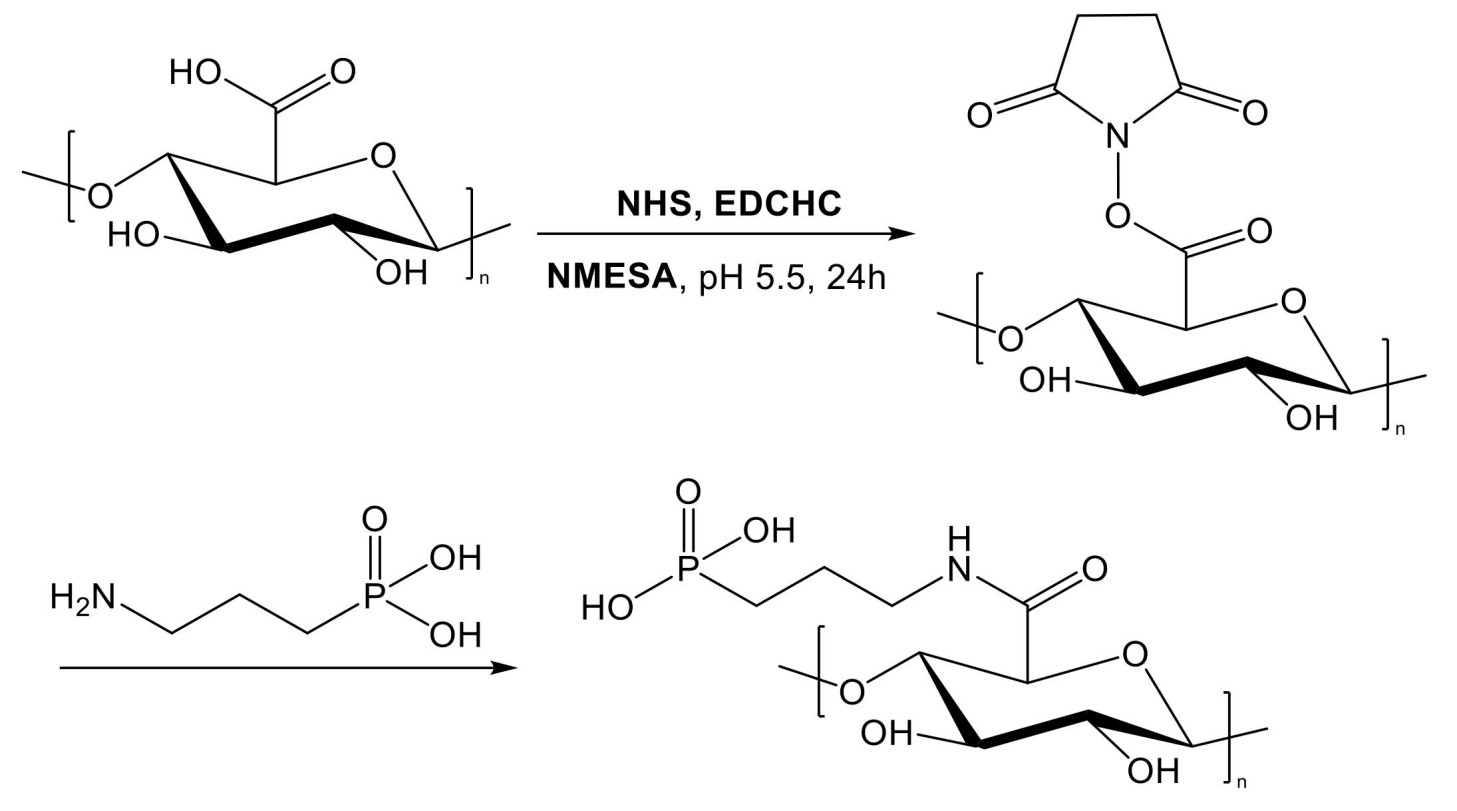
Reaction of TEMPO-oxidized cellulose nanofibrils (TO-CNF) with 3APPA to yield phosphorylated TO-CNF (PT-CNF).

TEMPO-oxidized cellulose nanofibrils (TO-CNF) were reacted with 3-aminopropylphosphoric acid (3APPA) under heterogeneous conditions, utilizing carbodiimide chemistry [52] after activation with 1-ethyl-3(3-dimethylaminopropyl)-1-carbodiimide hydrochloride (EDCHC) and N-hydroxysuccinimide (NHS) in 2-(N-morpholino) ethanesulfonic acid (NMESA) to yield phosphorylated TO-CNF (PT-CNF) [50]. Briefly, a suspension of 17.5 mg 3APPA in 4 mL water was added to 25 mL of a dispersion of TO-CNF (0.5% w/v) in 0.05 M NMESA buffer (1:1 M ratio between the carboxylic groups of TO-CNF and amino groups of 3APPA). The TO-CNF carboxylic groups were activated by the addition of the coupling reagents EDCHC and NHS in a 10:1 M ratio (based on carboxylic groups). The reaction mixture was incubated at 20 °C for 24 h under mild shaking, followed by 2 d dialysis against a cellulose membrane (1 kDa MWCO) to remove all non-reacted reagents. The reaction is displayed in Scheme 1.

### 2.3. Characterization of CNF

Freeze-dried TO-CNF and PT-CNF samples were analyzed by Fourier-transform infrared spectroscopy (FT-IR) using a PerkinElmer IR spectrophotometer (Waltham, MA, USA) with a Golden Gate Attenuated Total Reflectance (ATR, Specac Ltd, Orpington, UK) attachment. Spectra were recorded with 16 scans at a resolution of 4 cm^−1^ between 4000 and 650 cm^−1^ with the background air spectrum being subtracted.

The ζ-potential of both TO-CNF and PT-CNF dispersions (Milly-Q water, 0.005% w/v) was determined from electrophoretic mobility measurements using a Malvern Zeta Sizer (NanoZS, Malvern, UK). Average values were calculated from six individual measurements.

The charge content of PT-CNF was determined by conductometric titration. A sample of CNF (0.15 g dry weight) was suspended in water (total volume of 60 mL), a 5 mL 0.01 M NaCl solution was added, and the dispersion was magnetically stirred for 15 min. The pH was then adjusted to 2.8 by the addition of 0.1 M HCl. The titration was performed with a 0.04 M NaOH solution at a rate of 0.05 mL min^−1^ up to a pH of 11. During the titration, pH and conductivity were recorded. From these curves, the overall charge content was determined following the procedure published in [53].

### 2.4. Preparation of Nanopapers

Nanopapers were prepared following a previously reported method [47]. Homogeneous aqueous suspensions of CNF and PT-CNF, respectively, were prepared by adjusting the consistency to 0.2 wt % by the addition of deionized water and blending (Multiquick 5 MX 2050, Braun, Germany) for 2 min. This was followed by vacuum-filtration onto a cellulose filter paper (VWR 413, Lutterworth, UK) generating a filter cake that was wet-pressed under an applied load (10 kg) between blotting papers (3MM Chr VWR, Lutterworth, UK) for 5 min, thus absorbing excess water. These pre-dried filter cakes were then consolidated and dried in a hot-press (25-12-2H, Carver Inc., Wabash, IN, USA) (1 t, 1 h, 120 °C) by sandwiching them between fresh blotting papers and metal plates. PT-CNF nanopapers with grammages between 5 and 20 g m^−2^ (gsm) were prepared.

Nanopapers with enhanced porosity (PT-CNF in ethanol, hereafter EPT-CNF) were prepared from ethanol. First, a suspension of PT-CNF was prepared and filtered as described above. The resulting filter cake was then re-suspended in ethanol, blended and filtered again. The filter cake was then consolidated as described above to produce EPT-CNF nanopapers. EPT-CNF nanopapers with grammages between 3 and 14 gsm as well as one with 90 gsm were prepared. Different grammages for PT-CNF and EPT-CNF nanopapers, respectively, were chosen because it was expected that EPT-CNF nanopapers would have lower density/higher porosity. Thus, in order to obtain nanopapers of comparable thickness, a lower grammage was set for EPT-CNF nanopapers.

### 2.5. Characterization of Nanopapers

#### 2.5.1. Determination of Zeta-Potential of Nanopapers

The determination of the ζ-potential of EPT-CNF and PT-CNF nanopapers was performed using a SurPASS electrokinetic analyzer from Anton Paar (Graz, Austria) as a function of pH in an adjustable gap cell (gap width of 100 μm). A 1 mM KCl electrolyte solution was pumped through the cell while steadily increasing the driving pressure to 300 mbar. The pH of the electrolyte solution was controlled by titrating 0.05 mol L^−1^ HCl and 0.05 mol L^−1^ KOH, respectively. The measured streaming current was used to calculate the ζ-potential.

#### 2.5.2. Morphology of the Cellulose Nanopapers

The morphology of (E)PT-nanopapers was investigated using scanning electron microscopy (SEM). Specimens were cut from nanopapers, mounted onto aluminum stubs using carbon tabs, placed on a specimen holder, and directly sputter-coated (Leica EM QSG 100, Wieselaar, Germany) with a gold layer of approximately 10 nm. SEM images were taken on a Supra 55 VP at an accelerating voltage of 2 kV and a working distance of 7.2 to 7.3 mm.

#### 2.5.3. Specific Surface Area of Nanopapers by Nitrogen Sorption Experiments

Nitrogen sorption experiments with a surface area and porosity analyzer (TriStar II, Micromeritics, Neu-Purkersdorf, Austria) were performed to determine the specific surface area using the Brunauer–Emmett–Teller (BET) model. Prior to nitrogen adsorption experiments, samples were dried and degassed for 16 h at 120 °C (FlowPrep 060, Micromeritics, Norcross, GA, USA) by placing approximately 100 mg of sample inside a glass chamber and purging with nitrogen. Measurements of nitrogen adsorption isotherms were then performed in liquid nitrogen (77 K).

#### 2.5.4. Tensile Properties of Nanopapers

Tensile tests were performed at 25 °C and 50 % relative humidity using an Instron universal test frame (Model 5969 Dual Column Universal Testing System, Instron, Darmstadt, Germany) equipped with a 1 kN load cell. The tests were performed on rectangular (5 mm × 50 mm) specimens cut from the prepared papers. The thickness of each test specimen was measured at five different spots using a digital micrometer (705-1229, RS Components, Corby, UK) prior to each test. The crosshead displacement speed used was 1 mm min^−1^ and the gauge length was 20 mm.

#### 2.5.5. Thermal Degradation Behavior of the Papers

The thermal behavior of papers was investigated in nitrogen and air, respectively, by thermal gravimetric analysis (TGA) using a high resolution modulated TGA (Discovery TGA, TA Instruments, Eschborn, Germany). The samples of approximately 3 mg were heated from 30 to 650 °C at a heating rate of 10 °C min^−1^ and a gas flow rate of 25 mL min^−1^.

#### 2.5.6. Pure Water Permeance and Ion Adsorption Performance of Nanopapers

For the evaluation of the pure water permeance, discs of nanopapers with a diameter of 49 mm were placed on a porous stainless steel plate and installed in a stirred dead-end cell (Sterlitech HP4750, Kent, WA, USA). Deionized water was forced through the nanopapers (active filtration area of 1460 mm^2^) at 20 °C and a nitrogen head pressure of 0.1 or 0.2 MPa applied. The pure water permeance (L m^−2^ h^−1^ MPa^−1^) was calculated from the measured volume that permeated per unit area per unit time [44]. The permeances reported were stable within 1 % for 1 h.

To study the effectiveness of the nanopaper membranes for water hardness reduction, solutions of CaCl_2_ and MgCl_2_ were forced through the nanopapers. The solutions were prepared with 50 ppm (= 50 mg L^−1^, 1.25 mmol L^−1^ Ca^2+^, and 2.06 mmol L^−1^ Mg^2+^) each of Ca^2+^ and Mg^2+^ (in total 100 ppm), thereby imitating water with a high hardness of 18.5° dH. Permeate fractions were collected and diluted by a factor of 10 to enable analysis by flame atomic absorption spectroscopy (F-AAS) on an AAnalyst 200 (PerkinElmer). The mass of adsorbed calcium or magnesium ions per unit nanopaper area (mg m^−2^) was calculated from the volume of each permeate fraction and its concentration. Therefore, using the value for the active filtration area, the adsorption capacity, that is, the ratio of the mass of adsorbed ions to the total mass of CNF (mg g^−1^), was computed. For the EPT-CNF nanopaper with 90 gsm, a solution containing 80 ppm (2.0 mmol L^−1^) Ca^2+^ and 50 ppm (3.3 mmol L^−1^) Mg^2+^ was used. A 17 gsm PT-CNF nanopaper was tested with 50 ppm Ca^2+^ and 50 ppm Mg^2+^, respectively, alone to evaluate the adsorption capacity of the single cations without competition from the other ion.

## 3. Results and Discussion

### 3.1. Preparation and Characterization of CNF and Nanopapers

**Figure 1:**
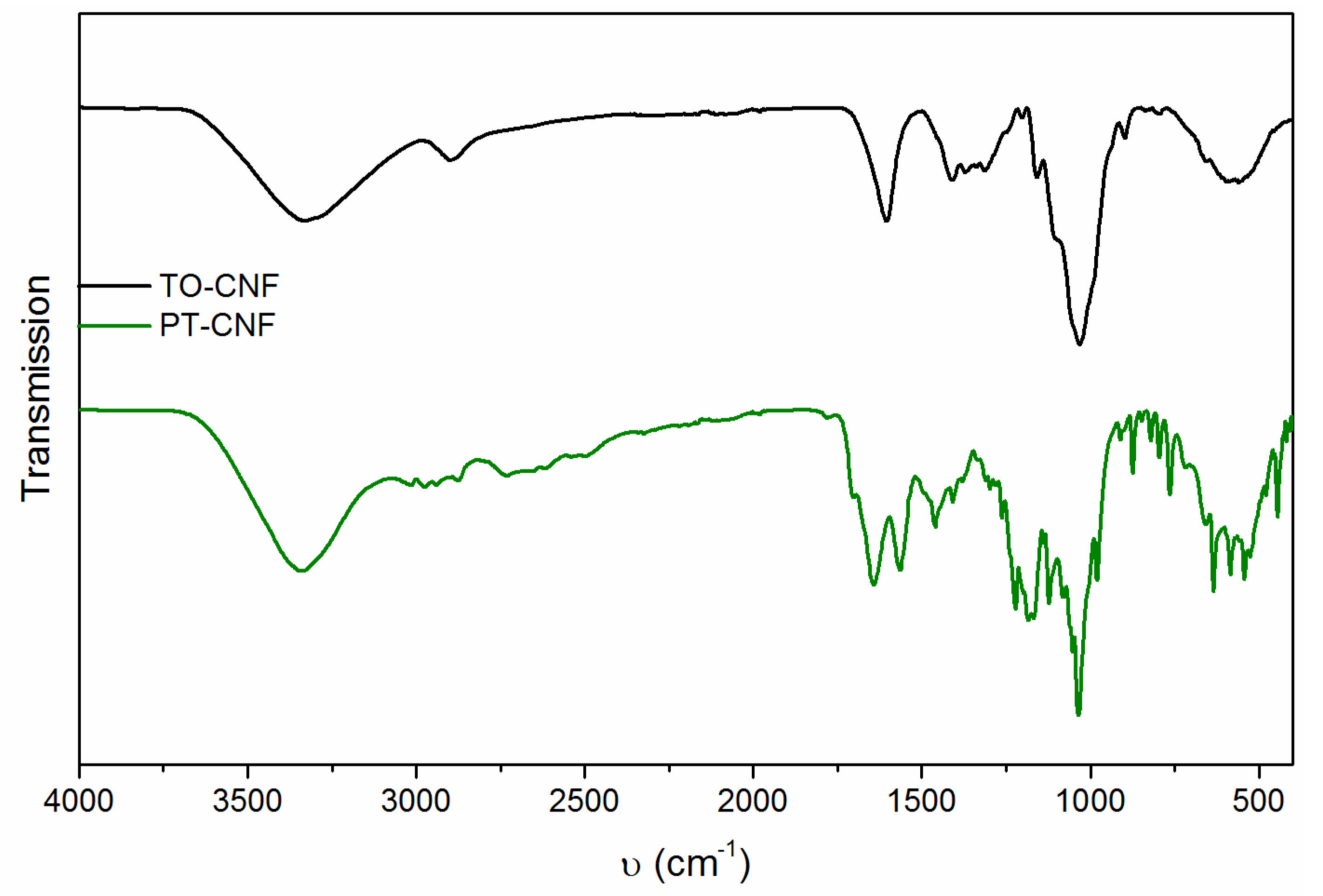
Fourier-transform infrared spectroscopy (FT-IR) spectra of (top, black) TO-CNF and (bottom, green) PT-CNF.

Success of the reaction to produce phosphorylated TO-CNF (PT-CNF) was confirmed by FT-IR spectroscopy, potentiometric titration, and electrophoretic (ζ-potential) measurements. The charge content of PT-CNF as determined by potentiometric titrations was 1.1 mmol g^−1^. This was slightly lower compared to the original TO-CNF (1.3 mmol g^−1^), which was expected as COOH-groups of TO-CNF were converted during the reaction via an uncharged intermediate. This was confirmed by the ζ-potential of PT-CNF, which was −12 mV and thus lower compared to TO-CNF (−18 mV). From the pH curves determined during conductometric titration, the presence of phosphonate groups could be verified: compared to pure TEMPO-CNF, for which a one-step neutralization process was found, a two-step process was observed for PT-CNF. The attachment of 3APPA onto TO-CNF was also confirmed by FT-IR spectroscopy (Figure 1). The presence of the COOH-group in the spectrum of TO-CNF was confirmed by the asymmetric vibration band at 1604 cm^−1^. In the spectrum of PT-CNF, this peak was shifted to 1643 cm^−1^ due to the coupling reaction resulting in an amide-bond. The band at 1566 cm^−1^ was related to N–H stretching. This was shifted as compared to pure 3APPA (1534 cm^−1^), which indicated a covalent reaction [50]. A band associated with the phosphonate group [54] was found at 1221 cm^−1^.

Nanopapers were manufactured using unmodified CNF as well as PT-CNF utilizing a papermaking process following a method previously published [44]. Furthermore, nanopapers were prepared from PT-CNF in ethanol suspensions (EPT-CNF). The aim of this study was to tailor the network structure in order to increase porosity (decrease density) that allows for higher water permeance. As an additional benefit of nanopapers with higher porosity, functional groups for the adsorption of calcium and magnesium ions are expected to be better accessible. The envelope density of the PT-CNF nanopapers was approximately 900 kg m^−3^, whereas the EPT-CNF nanopapers exhibited an envelope density of approximately 450 kg m^−3^. Taking into account a theoretical density of 1500 kg m^−3^ for cellulose [55], porosities of 40 % and 70 % for PT-CNF and EPT-CNF, respectively, were calculated. The repulsion between the less hydrophilic suspension medium ethanol and the hydrophilic nanofibrils is thought to contribute to these density differences [49]. Higher porosity of EPT-CNF nanopapers was also confirmed by nitrogen sorption experiments. For dense PT-CNF nanopapers, no specific surface area above the detection limit of our device could be determined; however, for EPT-CNF nanopapers (isotherm: Figure S1 in Supplementary Materials) the specific surface area was 69 m^2^ g^−1^.

**Figure 2:**
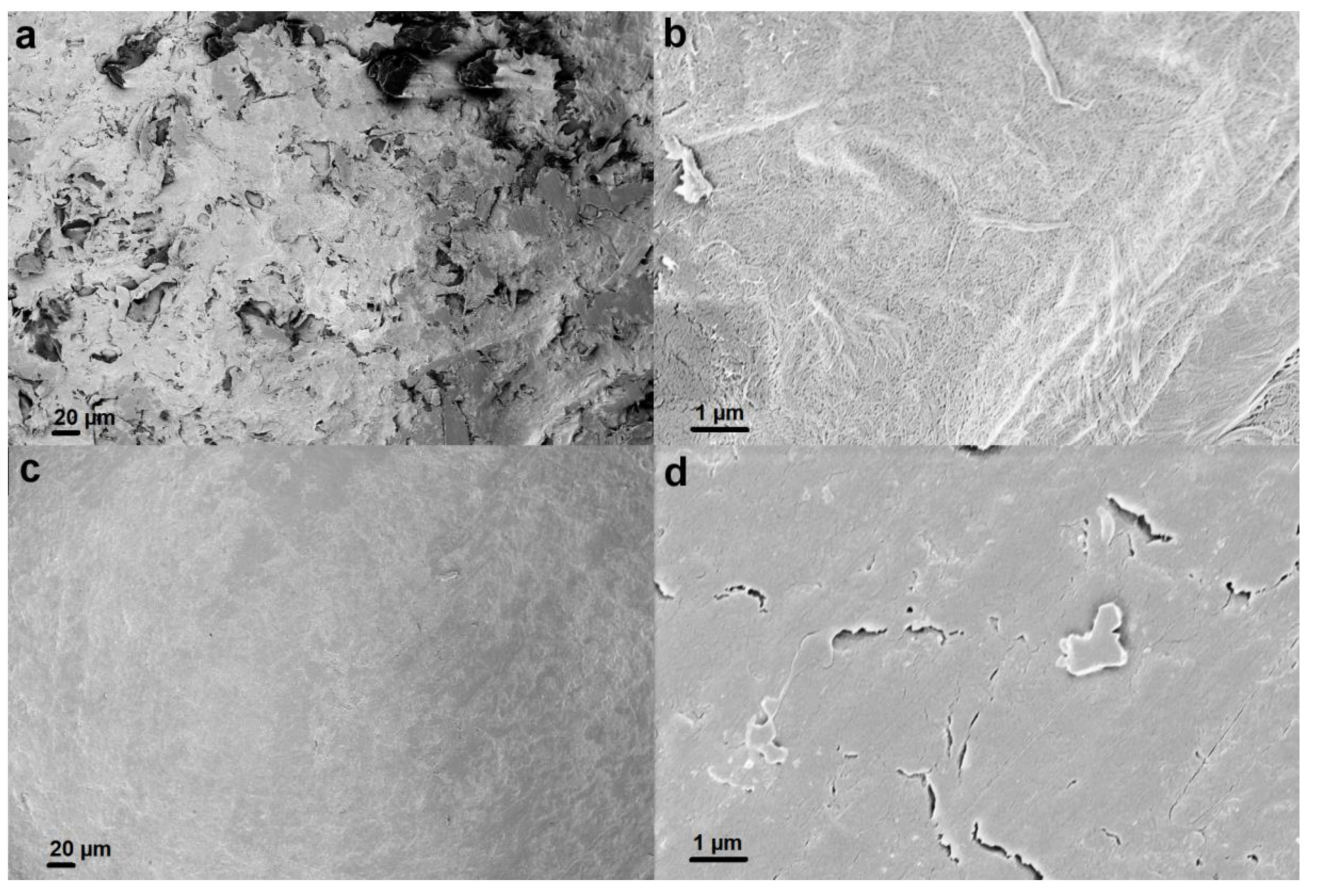
Scanning electron microscopy (SEM) images of EPT-CNF nanopapers at a magnification of (**a**) 250× and (**b**) 10,000× compared to PT-CNF nanopapers at a magnification of (**c**) 250× and (**d**) 10,000×.

SEM pictures of PT-CNF and EPT-CNF nanopapers are displayed in Figure 2. Already at low magnification (Figure 2a,c), obvious differences between the morphologies of PT-CNF and EPT-CNF can be seen. Whereas PT-CNF nanopapers (Figure 2c) have a smooth flat surface without detectable pores, EPT-CNF nanopapers (Figure 2a) have a very open, porous surface that should allow for high permeance. At a higher magnification, a homogeneous network of nanofibrils can be seen in PT-CNF nanopapers (Figure 2d). This network structure is present for EPT-CNF (Figure 2b) as well, but it appears to be more “open”, that is, fibrils are separated to a greater extent. The differences in the appearance of the nanopapers can be explained by the different densities and hence porosities that EPT-CNF and PT-CNF nanopapers exhibit due to the repulsion between hydrophilic CNF and less hydrophilic dispersion medium.

**Figure 3:**
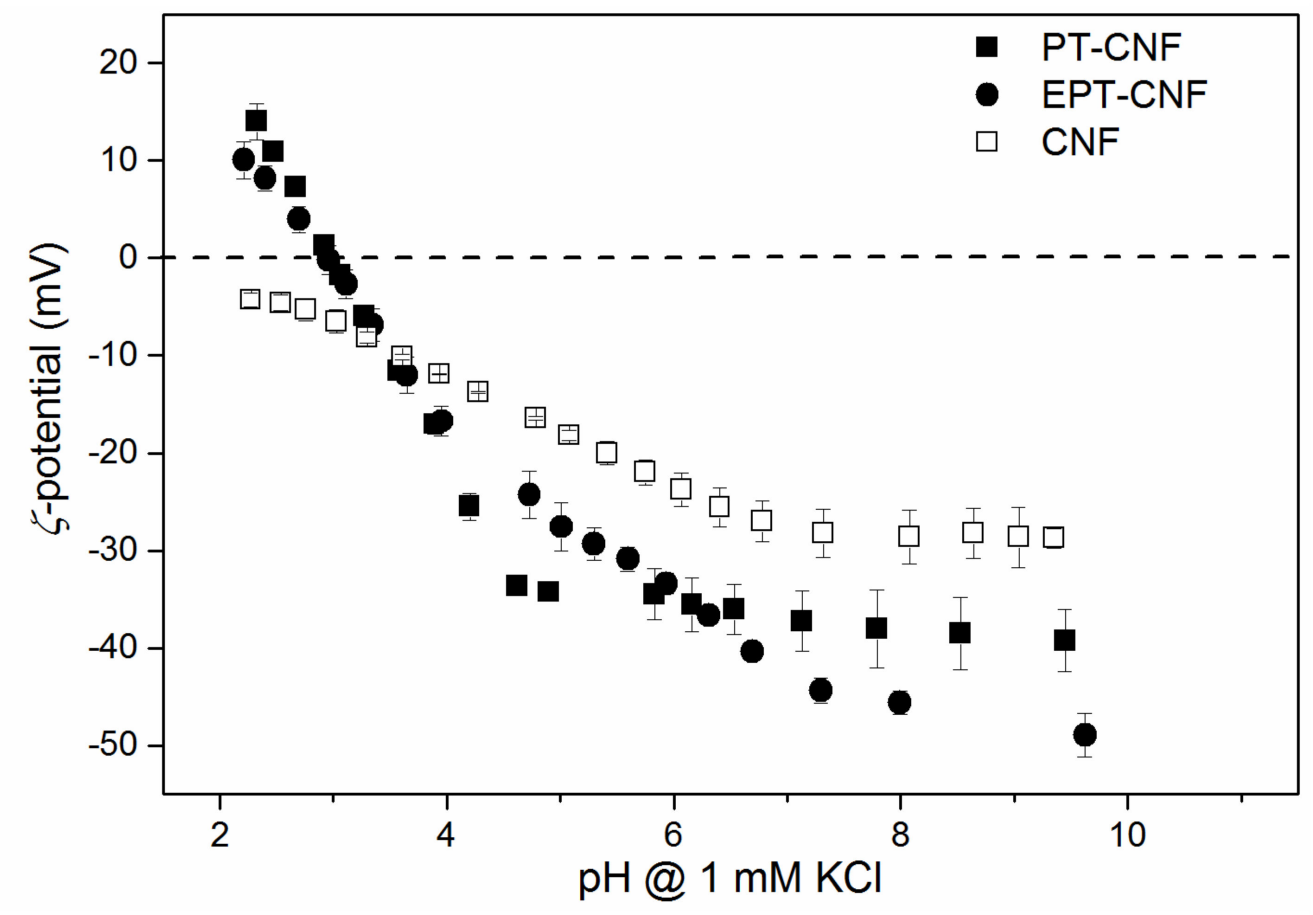
ζ-potential of (black square) PT-CNF, (black dot) EPT-CNF, and (empty square) unmodified nanopapers as a function of pH.

Figure 3 shows the ζ-potential of unmodified CNF, PT-CNF, and EPT-CNF nanopapers. Over the whole pH range analyzed, nanopapers from unmodified CNF had a negative ζ-potential between −2.5 and −21 mV, with a plateau in the basic pH range of approximately −21 mV. This indicates the presence of a significant amount of carboxyl groups (e.g., uronic acids) [56]. All dissociable functional groups are fully deprotonated and hence the surface is acidic [57]. Upon decrease of the pH, the ζ-potential increased for functional groups were protonated until the isoelectric point (IEP) was reached at approximately pH 2.1 (extrapolated). A significant difference in the ζ-potential between unmodified and (E)PT-CNF nanopapers was found. Both the introduction of carboxyl groups during TEMPO-oxidation as well as the attachment of phosphonate groups affected the surface charge. Phosphorylated TEMPO-CNF (PT-CNF) had a negative ζ-potential above pH 4, with a plateau at approximately −35 mV. The low value of the ζ-potential indicated both the presence of carboxyl groups and that of acidic phosphonate groups on the surface of the nanofibrils. Below pH 4, the negative ζ-potential decreased due to the protonation of functional groups until it reached a ζ-potential of +14 mV, passing the IEP at pH 3. This suggests that efficient adsorption of cations should occur above pH 3. At a pH below the IEP, the grafted phosphonate moieties were present in the form of hydrogen phosphonate groups, exhibiting positive ζ-potential [47]. Nanopapers prepared from suspensions of phosphorylated TEMPO-CNF in ethanol (EPT-CNF) rather than water had a slightly reduced plateau at a high pH of approximately −50 mV indicating a higher amount of deprotonated species being present, which was due to the higher porosity and thus the availability of functional groups on the surface of the EPT-CNF nanopapers. This reasoning is backed by the fact that the ζ-potential starts to increase already at mild acidic conditions (pH 6) due to the protonation of carboxyl and phosphonate groups. However, the IEP was passed at pH 3, just as was the case for PT-CNF nanopapers. This indicated that the same type of functional group was present but in different concentrations as shown by the lower plateau.

**Figure 4:**
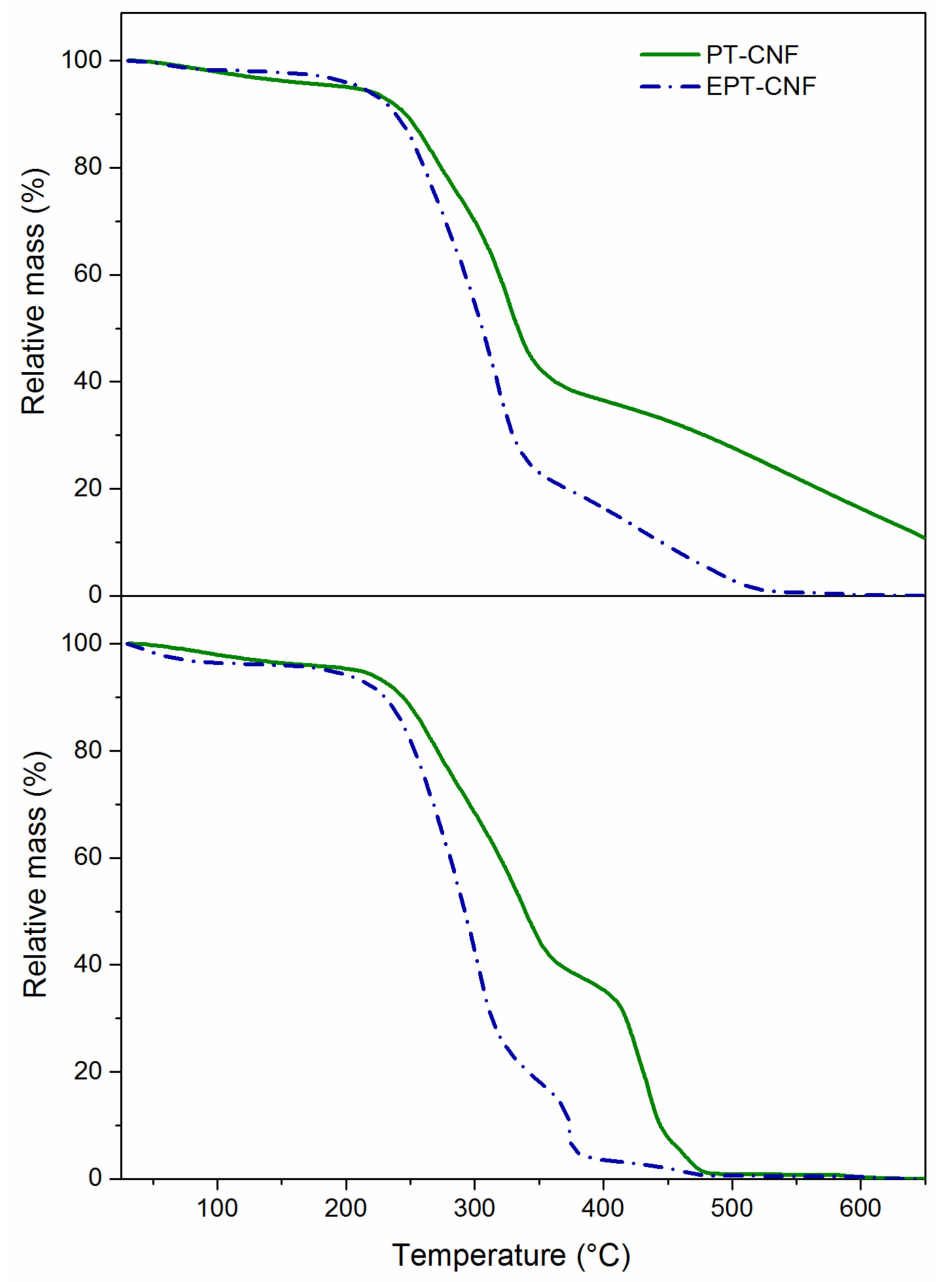
Thermograms of (green full line) PT-CNF and (blue dash-dotted line) EPT-CNF in (**top**) nitrogen and (**bottom**) air.

The tensile strength of the nanopapers (stress-strain-curves: Figure S2 in Supplementary Materials) was approximately 40 MPa for PT-CNF nanopapers and 25 MPa for EPT-CNF nanopapers, respectively, and thus more than 50 % higher for the denser PT-CNF nanopapers. This was in the range of common CNF filters/membranes and displayed the impact of higher porosity on the mechanical properties [46,47]. Nanopapers were further analyzed regarding their thermal stability by thermo-gravimetric analysis in both air and nitrogen atmosphere (Figure 4). The onset of degradation for both PT-CNF and EPT-CNF nanopapers was at approximately 250 °C in air and nitrogen, respectively. This proves that these nanopapers fulfill the requirements regarding their application in aqueous media. However, after the onset of degradation there is a clear distinction between the more porous EPT-CNF and the denser PT-CNF nanopapers. The mass loss at the primary degradation step is more significant in the case of the EPT-CNF nanopapers, which can be explained by their higher porosity and thus fewer interactions between CNF in more porous papers. This was found in both nitrogen and air atmosphere. A clear influence of the preparation medium was further observed in nitrogen. Whereas PT-CNF nanopapers were not degraded until 650 °C, EPT-CNF nanopapers were fully degraded already below 550 °C, which can be explained as well by lower interactions between nanofibrils that are farther separated in more porous nanopapers.

### 3.2. Permeance and Water Softening Performance of CNF Nanopapers

**Figure 5:**
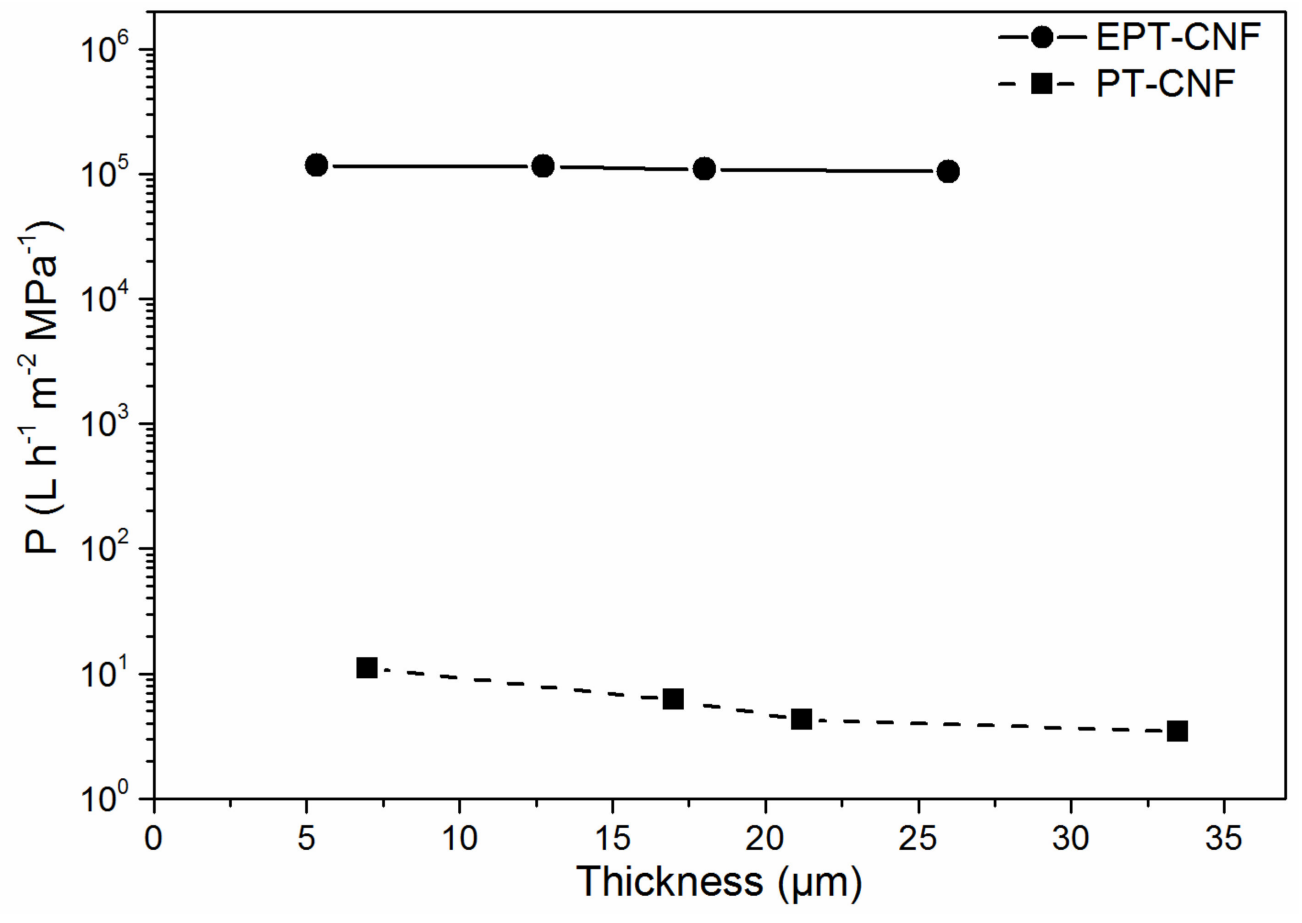
Permeance of (black squares, dashed line) PT-CNF and (black dots, full line) EPT-CNF nanopapers as a function of nanopaper thickness. The error of each data point is <1 %.

The big advantage of adsorption membranes over batch adsorbents is that they can be operated in a continuous fashion. So far the applicability of nanopapers to membrane applications has been limited due to their low permeance (P) caused by low porosity with pore sizes in the nanoscale [42,43,44]. The permeance of PT-CNF and EPT-CNF nanopapers is shown in Figure 5 as a function of their thickness. For both types of nanopapers, a logarithmic relationship between permeance and thickness was observed, as it is often the case for cellulose nanopapers [42,44]. PT-CNF nanopapers exhibited permeances between 3 and 11 L h^−1^ m^−2^ MPa^−1^, which is in the typical range of either modified or unmodified CNF nanopapers, respectively, as well as NF or RO membranes [36,47]. However, EPT-CNF nanopapers prepared from suspensions in ethanol had very high permeances above 100,000 L h^−1^ m^−2^ MPa^−1^, which is a factor of 10,000 higher than the permeance of PT-CNF nanopapers prepared from aqueous suspension. This huge increase can be explained by the much higher interconnected (Figure 2) porosity (70% vs. 40 %) of EPT-CNF nanopapers compared to PT-CNF nanopapers. Most importantly, by exhibiting this extremely high permeance already at pressures of 2 bar, EPT-CNF nanopapers outperform typical RO or NF membranes in terms of permeance [32,58].

**Figure 6:**
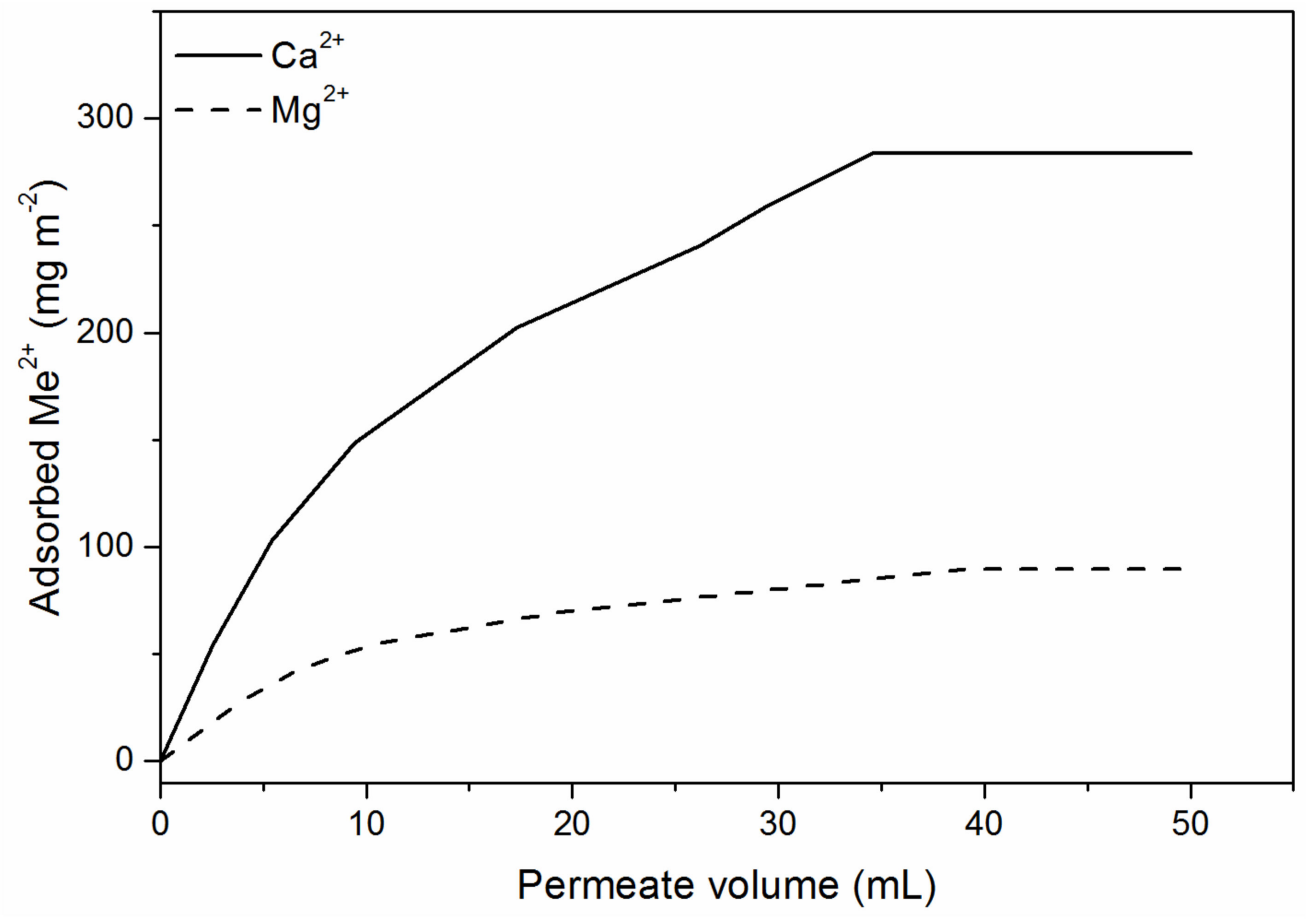
Adsorption of (full line) Ca^2+^ or (dashed line) Mg^2+^ ions onto PT-CNF nanopaper with 17 gsm per unit membrane area vs. permeate volume.

The water softening efficiency of nanopapers was assessed by dead-end filtration using model water containing 50 ppm Ca^2+^ (1.25 mmol L^−1^) and 50 ppm Mg^2+^ (2.06 mmol L^−1^). First, a PT-CNF nanopaper with 17 gsm was tested with aqueous solutions of either 50 ppm Ca^2+^ or Mg^2+^, but not in combination. In Figure 6, the amount of earth alkali ions Me^2+^ (i.e., Ca^2+^ or Mg^2+^) adsorbed per unit membrane area is plotted vs. the permeate volume. In particular, during the first stages of filtration a rapid removal of both ions was achieved. For the first 5 mL of testing (which corresponds to 3400 L water for 1 m^2^ of the membrane) of 0.25 mg Ca^2+^ and Mg^2+^, respectively, 0.15 mg and 0.05 mg, respectively, were removed. Thus, obvious differences were found regarding the adsorption of Ca^2+^ or Mg^2+^. Apparently, the affinity of PT-CNF nanopapers toward calcium ions was much higher compared to magnesium ions, even though a higher molar concentration of Mg ions was available. The adsorption capacity of PT-CNF nanopapers for Ca^2+^ and Mg^2+^ was 16.7 mg g^−1^ and 5.3 mg g^−1^, respectively. The result of a higher adsorption capacity of Ca^2+^ as compared to Mg^2+^ is in good accordance with literature data, which generally suggest a stronger affinity of Ca^2+^ to adsorbent materials than Mg^2+^ [14,15,16,59]. The differences can be explained by the higher hydration energy of magnesium and hence the greater volumetric dimensions of its hydrated ion in diluted aqueous solutions in comparison to calcium [14]. However, the non-hydrated ion radius of Ca^2+^ is bigger, thus enabling easier chelation between the two O atoms of the phosphonate group. This is also indicated by the binding ability between magnesium or calcium, respectively, and phosph(on)ate groups. Based on a different electronic structure, Mg^2+^ is capable of coordination to 6 O-ligands, whereas this number is 6 to 8 for Ca^2+^, depending on the ligand [60,61]. Hence, in particular in spatially restricted compounds, Ca^2+^ can be more easily chelated. The stronger bond between Ca^2+^ and phosphonate groups is also highlighted by the lower solubility of Ca_3_(PO_4_)_2_ as compared to Mg_3_(PO_4_)_2_.

**Figure 7:**
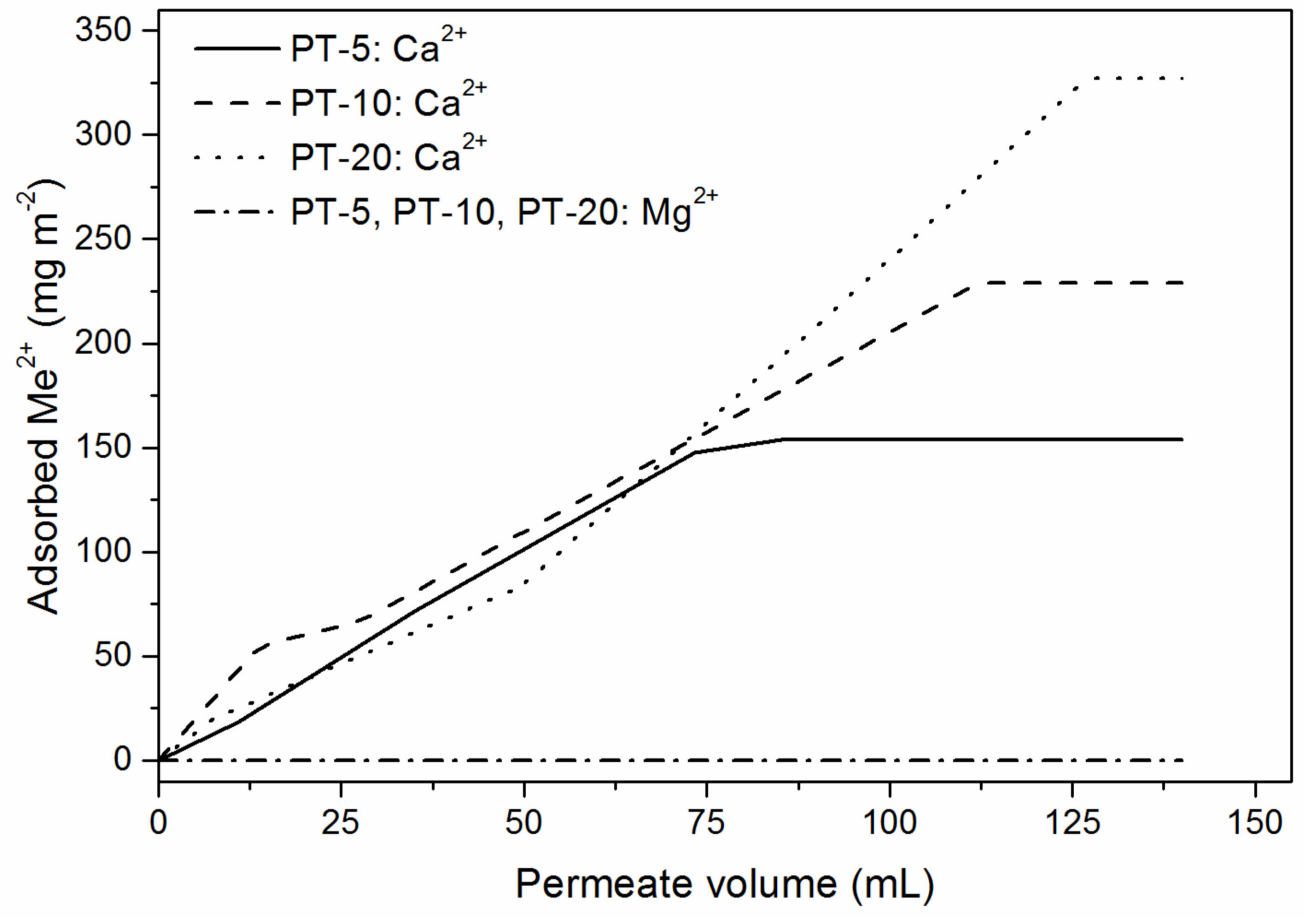
Adsorption of Mg^2+^ (dash-dotted line) and Ca^2+^ onto PT-CNF nanopapers with 5 (PT-5, full line), 10 (PT-10, dashed line) and 20 (PT-20, dotted line) gsm, respectively, per unit membrane area vs. permeate volume.

Tests with mixtures of Ca^2+^ and Mg^2+^ were performed with 50 ppm each of Ca^2+^ and Mg^2+^ in the same test solution. The results of these tests are shown in Figure 7 for PT-CNF nanopapers with 5 (PT-5), 10 (PT-10), and 20 (PT-20) gsm, respectively. No adsorption of Mg^2+^ could be detected at all for the PT-CNF nanopapers, which can be explained by the lower affinity of Mg^2+^ compared to Ca^2+^ to the active adsorbent agent and hence preferential adsorption of Ca^2+^. Furthermore, it was apparent that the slope of the Ca^2+^ adsorption graph was almost identical for all nanopapers, independently of their grammage. This suggests that there is no detectable difference in the rate at which the adsorption process proceeds on the surface of the nanopapers or in their bulk. However, the adsorption of Ca^2+^ on the surface of the nanopapers apparently contributed more to the total adsorption capacity because the increased adsorption capacity did not linearly correlate with the total amount of carboxyl and phosphonate groups present in the nanopaper, which increased linearly with increasing grammage. It was found that the heavier the nanopapers and thus the more functional groups were available, the more Ca^2+^ could be adsorbed. Therefore, for the PT-CNF paper with 5 gsm, approximately 150 mg m^−2^ were found, whereas for the 20 gsm paper this value was approx. 330 mg m^−2^. However, if the adsorption capability was equal for functional groups in the bulk of the nanopaper and on their surface, a value of 600 mg m^−2^ for the 20 gsm nanopaper should be expected. The lower value showed that functional groups on the surface of the nanopapers are easier accessible for adsorption of ions than functional groups in the bulk of the nanopaper due to the high density and hence tight packing of the fibrils. This is in good agreement with previous findings [36,47,48]. Accordingly, adsorption capacities were found to be higher for nanopapers with lower grammage (Table 1). If the availability of functional groups on the surface of the nanopapers was the same as in their bulk, the adsorption capacities (mg g^−1^) should be equal independently of the nanopaper grammage. As this was not the case, it can be concluded that the surface of the nanopapers contributed more strongly to the adsorption of metal ions. The reason for this phenomenon is thought to be associated with the overlap and eventual collapse of the electrochemical double layer around the fibrils when they are in close proximity [62]. In dense nanopapers in which the fibrils are tightly packed, this collapse of the double layer is present to a much greater extent compared to nanopapers in which the fibrils are less tightly packed. In general, the adsorption capacities found were similar to adsorption capacities presented for modified cellulose nanopapers toward nitrate or heavy metal ions [36,47]. Furthermore, as no Mg^2+^ adsorption was detected, the adsorption capacity for Ca^2+^ from the mixture was almost identical to the value determined when only calcium containing water was forced through the nanopaper, as shown above. However, it has to be noted that in the presence of Mg^2+^ the rate of adsorption of Ca^2+^ was lower, which refers to slower kinetics of Ca^2+^ adsorption when Mg^2+^ is present.

**Figure 8:**
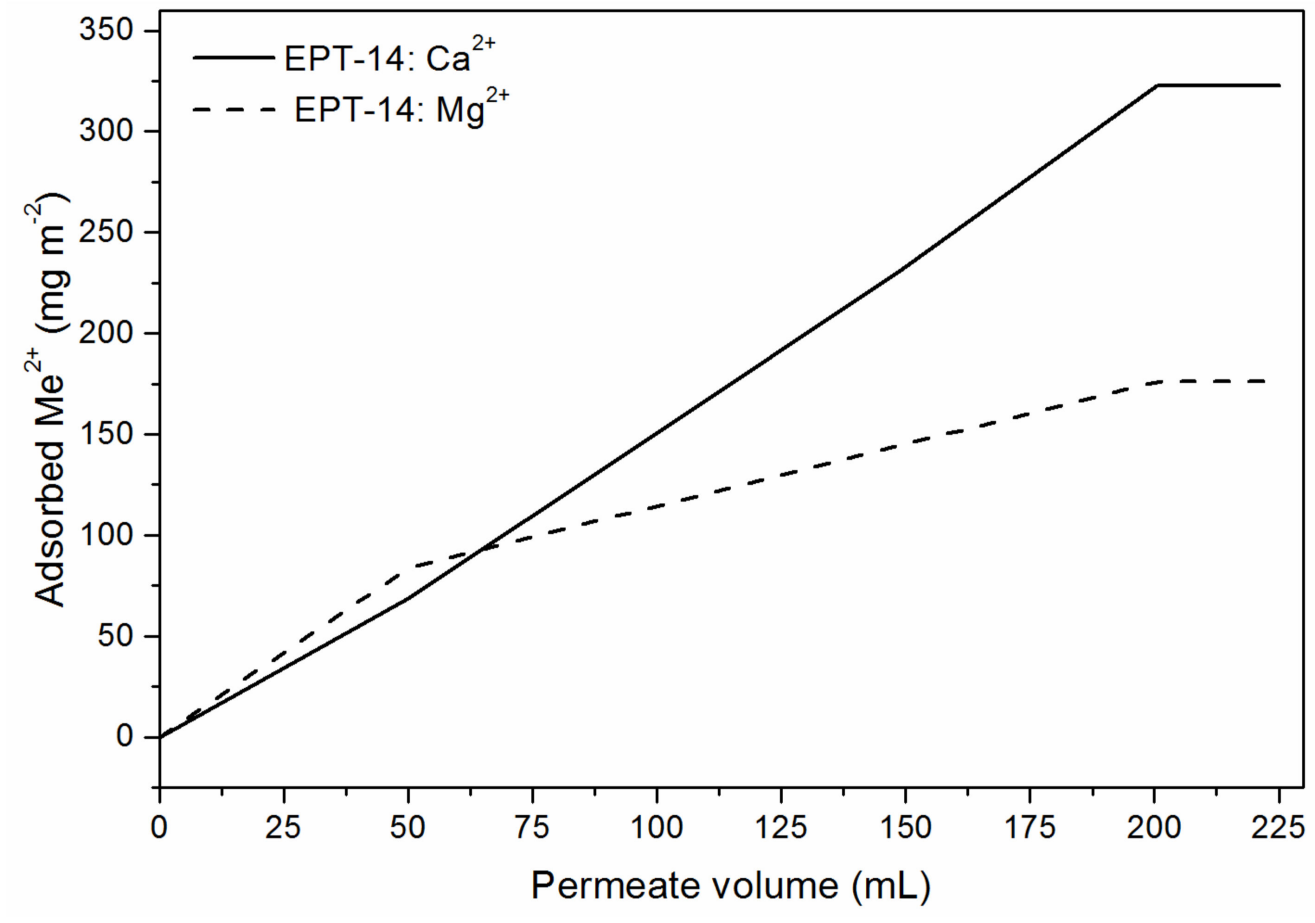
Adsorption of (full line) Ca^2+^ and (dashed line) Mg^2+^ ions onto an EPT-CNF nanopaper 14 gsm per unit membrane area vs. permeate volume.

The adsorption capacities found would already be appreciable; however, in combination with moderate permeances (3 to 11 L h^−1^ m^−2^ MPa^−1^), the overall performance was rather low. Therefore, we aimed to improve the performance of the nanopapers, primarily to increase the permeance but also the adsorption capacity. The approach which we used was based on the assumption that nanopapers produced from CNF suspensions in organic liquids [49] have a much higher porosity as compared to nanopapers prepared from aqueous suspensions. Success of this method is shown above; we demonstrated that the permeance of nanopapers can be increased by a factor of more than 10,000. Moreover, the higher porosity should result not only in an increased permeance but also in an enhanced adsorption capacity as a higher fraction of functional groups is now exposed within the bulk of the nanopaper and available for adsorption of metal ions. As already shown before [47,48], and also within this study, adsorption preferably takes place on the surface of nanopapers and is less favored within the nanopaper bulk. Within dense nanopapers the adsorption of ions is restricted due to collapse of the electrochemical double layer around tightly packed fibrils [62]. If, on the other hand, the density of the nanopapers is lowered (i.e., the porosity is higher), this increases the functionality of adsorption groups in the bulk of the nanopaper. To test this hypothesis, adsorption experiments were performed with EPT-CNF nanopapers. Specifically, the results of these experiments are shown in Figure 8 for EPT-CNF nanopapers with 14 gsm.

In contrast to PT-CNF nanopapers prepared from aqueous suspension, EPT-CNF nanopapers were capable of adsorbing not only Ca^2+^ but also Mg^2+^ because, overall, more functional groups were available. This was due to the greater accessibility of functional groups in the bulk of the nanopapers, enabling preferential adsorption not only of Ca^2+^ but also of Mg^2+^. Still, the amount of magnesium ions adsorbed (180 mg m^−2^, 7.4 mmol m^−2^) was lower compared to 320 mg m^−2^ (8 mmol m^−2^) for calcium. However, initially more Mg^2+^ was adsorbed, which must have been partly replaced by Ca^2+^, and differences were much smaller than for the dense PT-CNF nanopapers. This was thought to be due to the higher abundance of active adsorption sites in the more porous EPT-CNF nanopapers. In terms of adsorption capacity, this amounts to 22.6 mg g^−1^ (0.56 mmol g^−1^) Ca^2+^ and 12.3 mg g^−1^ (0.51 mmol g^−1^) Mg^2+^, respectively, for 14 gsm EPT-nanopapers. However, for lower grammages very high adsorption capacities of up to 91 mg g^−1^ (2.3 mmol g^−1^) were found (Table 2). This is on par with or even exceeds the level of state-of-the-art batch absorbents [2,15,63,64] but with the advantage of a rapid, continuous process. Eventually, in particular for very thin nanopapers, even more Mg^2+^ was adsorbed than Ca^2+^ on a molar base. Due to much more space available between nanofibrils in porous nanopapers prepared from ethanol, the spatial restriction that preferred chelation of Ca^2+^ was no longer overwhelming and the smaller radius of non-hydrated Mg^2+^ enabled better adsorption.

Thus, we demonstrated that highly porous (EPT-)nanopapers are a true alternative, in terms of adsorption performance combined with very high permeance, to synthetic ion-exchange resins. These are commonly operated at approximately 5 bar, whereas the nanopapers can be operated already at only 1 bar. The higher adsorption capacities of nanopapers with lower grammage demonstrate that, even for high porosity EPT-nanopapers in which utilization of functional groups within the bulk of the nanopaper is improved, adsorption on the surface of the nanopaper is the major contributor of ion adsorption.

**Figure 9:**
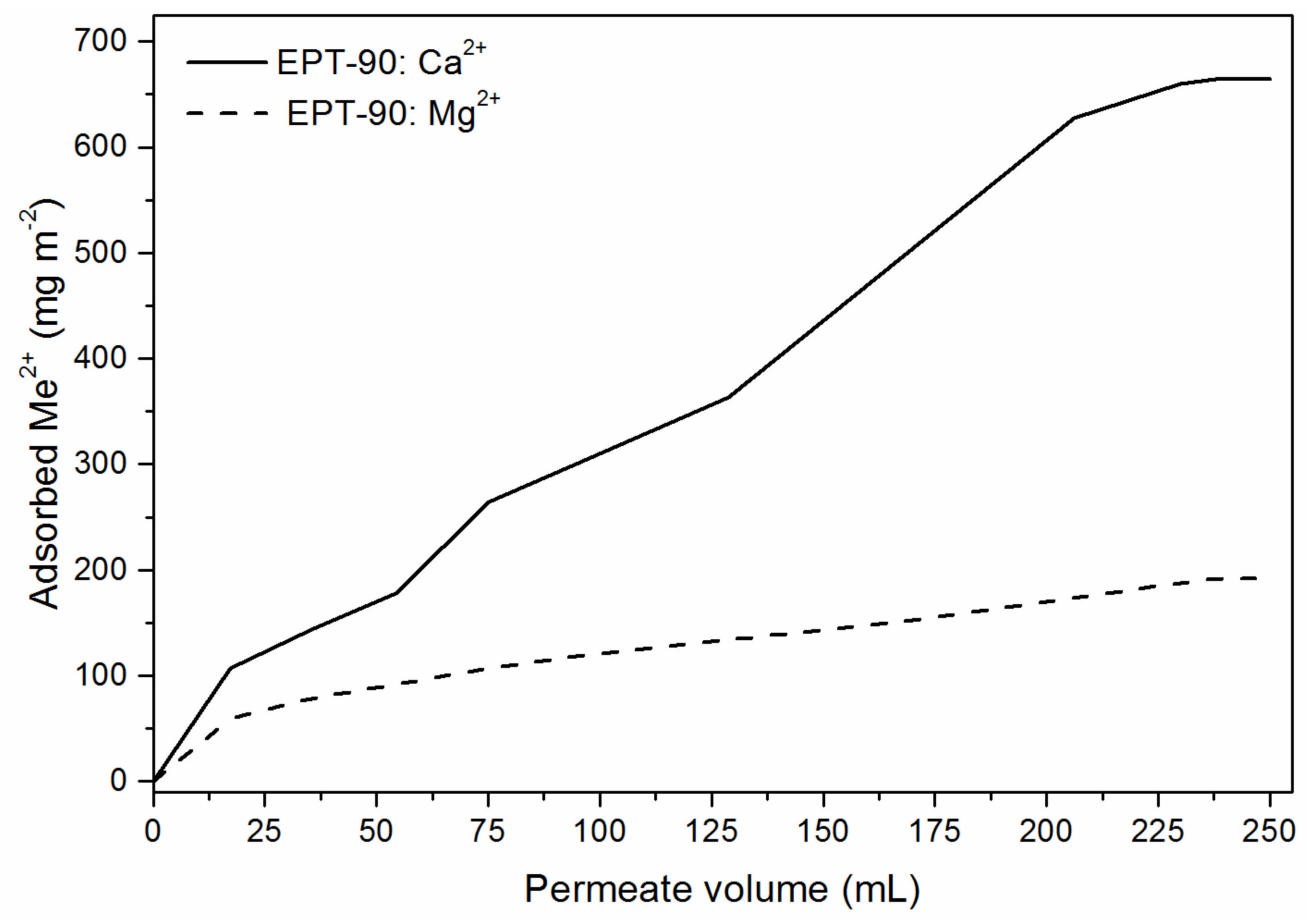
Adsorption of (full line) Ca^2+^ and (dashed line) Mg^2+^ onto a 90 gsm EPT-CNF nanopaper per unit membrane area vs. permeate volume.

Finally, a very thick EPT-CNF nanopaper with 90 gsm was tested with a solution of 80 ppm Ca^2+^ and 50 ppm Mg^2+^. The higher Ca^2+^ concentration was used to mimic natural water more closely in which Ca^2+^ is often present in higher mass concentrations than Mg^2+^ [65]. The results of these experiments are shown in Figure 9. Confirming the findings presented above for EPT-nanopapers with lower grammage, Ca^2+^ was preferentially adsorbed on the nanopaper, with the biggest amount of Ca^2+^ adsorbed per unit area (660 mg m^−2^) found within this study. A lower value was found for Mg^2+^, comparable to the nanopapers of lower mass, with 190 mg m^−2^. These values correspond to an adsorption capacity of 7.4 and 2.1 mg g^−1^ for Ca^2+^ and Mg^2+^, respectively. These relatively low values lent further support to the hypothesis of preferential adsorption occurring on the surface of the nanopapers compared to the bulk. Still, within EPT-CNF nanopapers, bulk adsorption also contributes significantly to the overall adsorption capacity, as was shown by comparing the adsorption per unit area: 660 mg m^−2^ for 90 gsm vs. 320 mg m^−2^ for 14 gsm nanopapers. Nevertheless, thinner nanopapers have a higher adsorption capacity (mg g^−1^), as compared in Table 2.

Adsorption capacities achieved were compared to recently published data (Table 3), which show that the current approach can easily compete with state-of-the-art solutions and for the most part outperform them.

## 4. Conclusions

Cellulose nanopapers were prepared via an easy papermaking process from phosphorylated TEMPO-oxidized cellulose nanofibrils with a total charge content of 1.1 mmol g^−1^. Measured ζ-potentials confirmed the increased negative surface charge due to the introduction of carboxyl and phosphonate groups compared to unmodified nanopapers. Nanopapers from both aqueous suspensions as well as from suspensions in organic media (i.e., ethanol) were manufactured to study the influence of the dispersion media on the porosity of the resulting nanopapers. The suspension of cellulose nanofibrils in ethanol led to the formation of a paper network with much higher porosity, resulting in much higher permeances, which were a factor of more than 10,000 higher compared to nanopapers prepared from aqueous suspensions. For nanopapers prepared from aqueous suspensions, an adsorption capacity for Ca^2+^ of approximately 20 mg g^−1^ (5.3 mg g^−1^ for Mg^2+^) was found, whereas nanopapers prepared from ethanol suspensions had Ca^2+^ adsorption capacities of up to 91 mg g^−1^ (69 mg g^−1^ for Mg^2+^). The higher adsorption capacity can be explained by a much better accessibility of functional groups within the bulk of the nanopapers due to higher porosity and more available functional groups on the interior surfaces within the nanopaper. Thus, together with the very high permeance, a very good overall performance of these nanopapers in water softening applications was achieved.

## Figures and Tables

**Table 1:** Adsorption capacities of Ca^2+^ and Mg^2+^ for PT-CNF nanopapers with 5, 10, and 20 gsm, respectively.

Grammage (gsm)	Adsorption Capacity (mg g^−1^)		Adsorption per Unit Area (mg m^−2^)	
	Ca^2+^	Mg^2+^	Ca^2+^	Mg^2+^
5	24.5	-	150	-
10	21.2	-	230	-
20	17.4	-	330	-

**Table 2:** Adsorption capacities of Ca^2+^ and Mg^2+^ for EPT-CNF nanopapers with 3, 9, and 14 gsm, respectively.

Grammage (gsm)	Adsorption Capacity (mg g^−1^)		Adsorption Capacity (mmol g^−1^)		Adsorption per Unit Area (mg m^−2^)	
	Ca^2+^	Mg^2+^	Ca^2+^	Mg^2+^	Ca^2+^	Mg^2+^
3	90.8	68.9	2.26	2.84	270	210
9	35.7	25.1	0.89	1.03	320	230
14	22.6	12.3	0.56	0.51	320	180

**Table 3:** Adsorption capacities for Ca^2+^ and Mg^2+^ compared to the literature.

Material	Reference	Adsorption Capacity (mg g^−1^)	
		Ca^2+^	Mg^2+^
Phosphorylated TEMPO-CNF	this study	91	69
Mesoporous LTA zeolite	[7]	108	28
Carboxylated polyacrylic resin	[63]	51	-
Carbon nanotube sheets	[2]	80	94
Electrospun anionic cellulose nanofibers	[64]	58	66
Sugarcane bagasse	[66]	14	16
EDTA dianhydride grafted sugarcane bagasse	[66]	46	23
Natural pumice	[15]	57	45
Modified pumice	[15]	62	56

## Supplementary Materials

The following are available online at http://www.mdpi.com/2079-4991/9/2/136/s1, Figure S1: Nitrogen sorption isotherm for EPT-CNF nanopapers; Figure S2: Exemplary stress-strain-curves for PT-CNF and EPT-CNF nanopapers.
