# Supplementary material for: Rapid Water Softening with TEMPO-Oxidized/Phosphorylated Nanopapers
Source: Nanomaterials (Basel). 2019 Jan 22;9(2):136. doi: 10.3390/nano9020136 (PMC6409817; doi:10.3390/nano9020136)
Supplement: Supplementary file 1 [file nanomaterials-09-00136-s001.pdf]

nanomaterials

Supporting Information for

Rapid Water Softening with TEMPO-oxidized/phosphorylated Nanopapers

Andreas Mautner [1,2,*], Thawanrat Kobkeatthawin [1,3], Florian Mayer [1], Christof Plessl [4], Selestina Gorgieva [5], Vanja Kokol [5], and Alexander Bismarck [1,2]

1 Polymer & Composite Engineering (PaCE) Group, Institute of Materials Chemistry & Research, University of Vienna, 1090 Vienna, Austria; Thawanrat_KK@hotmail.com (T.K.); f.mayer@univie.ac.at (F.M.); alexander.bismarck@univie.ac.at (A.B.)
2 Polymer & Composite Engineering (PaCE) Group, Department of Chemical Engineering, Imperial College London, SW7 2AZ London, United Kingdom
3 Department of Chemistry and Center of Excellence for Innovation in Chemistry, Faculty of Science, Prince of Songkla University, Songkhla 90110, Thailand
4 Institute of Inorganic Chemistry, University of Vienna, 1090 Vienna, Austria; christof.plessl@univie.ac.at
5 Institute for Engineering Materials and Design, Faculty of Mechanical Engineering, University of Maribor, 2000 Maribor, Slovenia; selestina.gorgieva@um.si (S.G.); vanja.kokol@um.si (V.K.)
* Correspondence: andreas.mautner@univie.ac.at; Tel.: +43-1-4277-71324

1. Nitrogen sorption experiments of EPT-CNF nanopapers

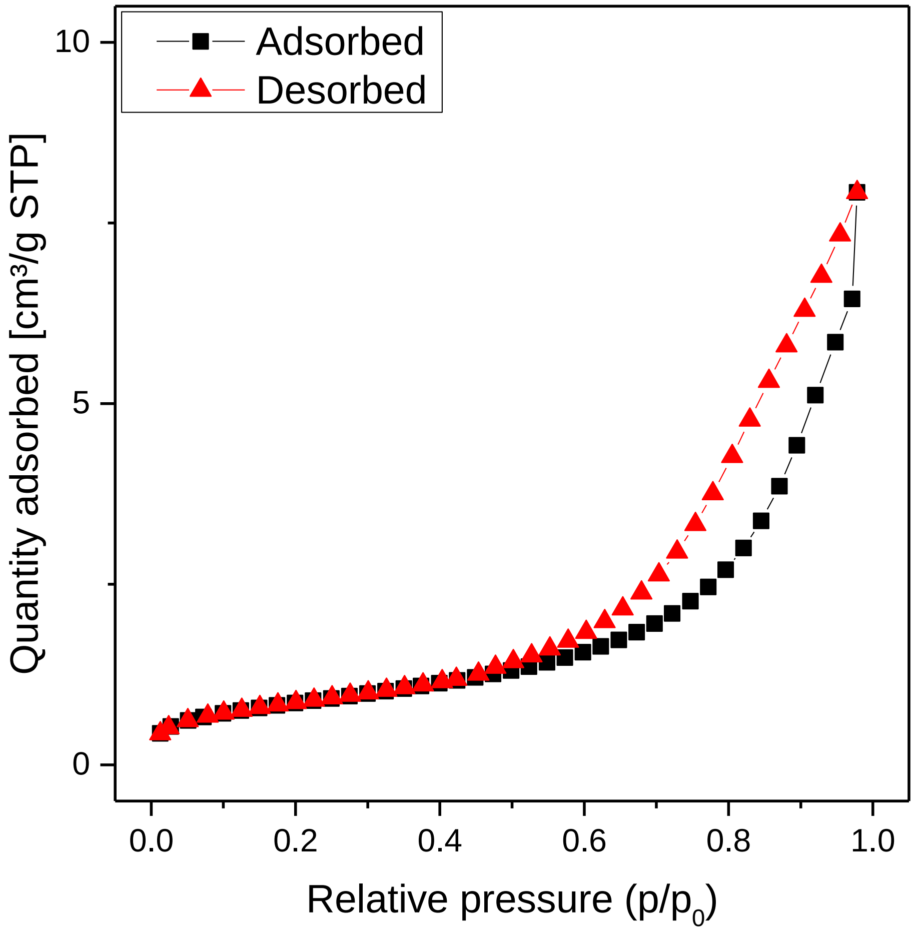

Figure S1. Nitrogen sorption isotherm for EPT-CNF nanopapers.

2. Tensile tests of PT-CNF and EPT-CNF nanopapers

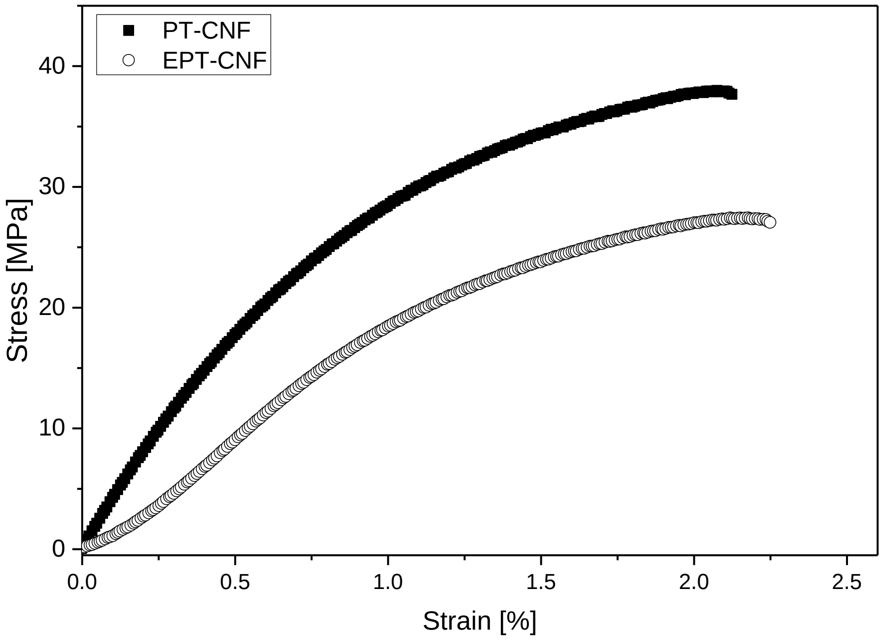

Figure S2. Exemplary stress-strain-curves for PT-CNF and EPT-CNF nanopapers.

© 2019 by the authors. Submitted for possible open access publication under the terms and conditions of the Creative Commons Attribution (CC BY) license (http://creativecommons.org/licenses/by/4.0/).
